# Cytotoxicity of Cyclodipeptides from *Pseudomonas aeruginosa* PAO1 Leads to Apoptosis in Human Cancer Cell Lines

**DOI:** 10.1155/2015/197608

**Published:** 2015-03-02

**Authors:** Dolores Vázquez-Rivera, Omar González, Jaquelina Guzmán-Rodríguez, Alma L. Díaz-Pérez, Alejandra Ochoa-Zarzosa, José López-Bucio, Víctor Meza-Carmen, Jesús Campos-García

**Affiliations:** ^1^Instituto de Investigaciones Químico Biológicas, Universidad Michoacana de San Nicolás de Hidalgo (UMSNH), Edificio B-3, Ciudad Universitaria, 58030 Morelia, MICH, Mexico; ^2^Centro Multidisciplinario de Estudios en Biotecnología, Facultad de Medicina Veterinaria y Zootécnia, UMSNH, 58893 Morelia, MICH, Mexico

## Abstract

*Pseudomonas aeruginosa* is an opportunistic pathogen of plants and animals, which produces virulence factors in order to infect or colonize its eukaryotic hosts. Cyclodipeptides (CDPs) produced by *P. aeruginosa* exhibit cytotoxic properties toward human tumor cells. In this study, we evaluated the effect of a CDP mix, comprised of cyclo(L-Pro-L-Tyr), cyclo(L-Pro-L-Val), and cyclo(L-Pro-L-Phe) that were isolated from *P. aeruginosa*, on two human cancer cell lines. Our results demonstrated that the CDP mix promoted cell death in cultures of the HeLa cervical adenocarcinoma and Caco-2 colorectal adenocarcinoma cell lines in a dose-dependent manner, with a 50% inhibitory concentration (IC_50_) of 0.53 and 0.66 mg/mL, for HeLa and Caco-2 cells, respectively. Flow cytometric analysis, using annexin V and propidium iodide as apoptosis and necrosis indicators, respectively, clearly showed that HeLa and Caco-2 cells exhibited apoptotic characteristics when treated with the CDP mix at a concentration <0.001 mg/mL. IC_50_ values for apoptotic cells in HeLa and Caco-2 cells were 6.5 × 10^−5^ and 1.8 × 10^−4^ mg/mL, respectively. Our results indicate that an apoptotic pathway is involved in the inhibition of cell proliferation caused by the *P. aeruginosa* CDP mix.

## 1. Introduction

*Pseudomonas aeruginosa* colonizes many biological environments, such as soil, plants, and animal tissue, being an important pathogen involved in opportunistic infections in humans [1] and a major cause of nosocomial infections [2]. Several mechanisms for driving infection in the host have been attributed to* P. aeruginosa*, and, among these, the production of toxins, adhesins, pyocyanin, and other virulence factors plays an important role in infecting different hosts, from plants to animals [3, 4].* P. aeruginosa* produces and senses N-acyl-L-homoserine lactones (AHLs) for cell-to-cell communication via a regulatory mechanism known as quorum sensing (QS), which links the perception of bacterial cell density to gene expression. QS coordinates many physiological processes, such as symbiosis, production of virulence factors, resistance to oxidative stress, antibiotic resistance, motility, biofilm formation, and the progression of* P. aeruginosa* infection in animals [5, 6].

The cyclodipeptides (CDPs) cyclo(D-Ala-L-Val) and cyclo(L-Pro-L-Tyr) have been identified in* P. aeruginosa* cultures, which led to the proposition that CDPs have the ability to inhibit the activity of regulatory LuxR-type proteins that are involved in AHL-dependent QS signaling. This in turn led to the proposition that CDPs and their derivatives, the diketopiperazines (DKPs), represent a new class of QS signals and that they could potentially act as interkingdom signals. However, the mechanism of action and physiological relevance of CDPs are poorly understood [7, 8].

**Figure 1:**
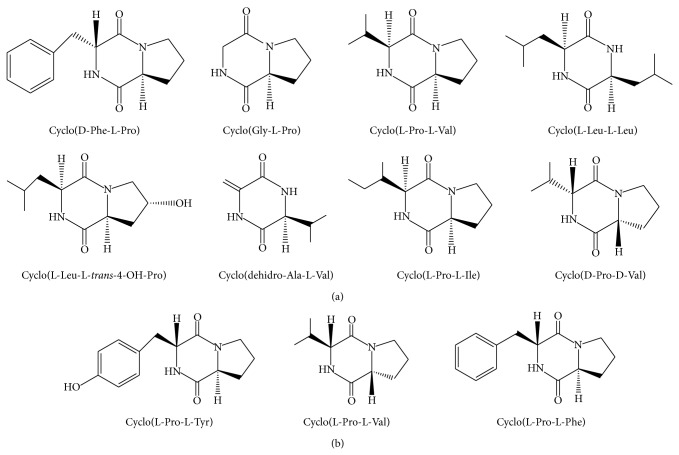
Chemical structures of cyclodipeptides (CDPs) from bacteria. (a) Structures of CDPs synthesized by some bacterial species, modified from [9, 10]. (b) CDPs isolated from* Pseudomonas aeruginosa* strain PAO1. Isolation of CDPs was carried out according to the previously reported protocol [12]. A mixture of CDPs, mainly comprised of cyclo(L-Pro-L-Tyr), cyclo(L-Pro-L-Val), and cyclo(L-Pro-L-Phe) in a 1 : 1 : 1 molar ratio, was used in this study.

DKPs are cyclized molecules comprising two amino acids bound by two peptide bonds; they are produced by a wide range of organisms, from bacteria to fungi and animals (Figure 1(a)) [9, 10]. DKPs belong to the nonribosomal peptides that are synthesized in microorganisms by a multifunctional assembly of enzymes known as nonribosomal peptide synthases [10] and by CDP synthases, another kind of enzymes that utilizes aminoacylated transfer RNAs as substrates instead of free amino acids [11].

CDPs are structurally diverse, and they have been implicated in multiple functions; the CDPs cyclo(D-Ala-L-Val) and cyclo(L-Pro-L-Tyr) have been identified as a new class of QS autoinducers in* Pseudomonas* strains, based on their ability to activate AHL-dependent biosensors [12–14]. The CDP cyclo(L-Phe-L-Pro) isolated from* Lactobacillus plantarum* exhibited an antifungal effect against* Fusarium sporotrichioides* and* Aspergillus fumigatus* [15], while the CDPs cyclo(L-Leu-L-Pro), cyclo(L-Phe-L-Pro), cyclo(L-Val-L-Pro), cyclo(L-Trp-L-Pro), and cyclo(L-Leu-L-Val) isolated from the deep-sea bacterium* Streptomyces fungicidicus* showed antifouling effects [16]. Moreover, synthetic CDPs such as cyclo(Phe-Pro) induced apoptosis in the HT-29 colon cancer cell line [17], and cyclo(L-Cys-L-Leu) exhibited potential for scavenging free radicals [18]. Recently, it was reported that* P. aeruginosa* is capable of interacting with the plant* Arabidopsis thaliana* via the secretion of CDPs such as cyclo(L-Pro-L-Tyr), cyclo(L-Pro-L-Val), and cyclo(L-Pro-L-Phe), appearing to mimic the biological role of auxin, a natural plant hormone [12] (Figure 1(b)). In* Staphylococcus aureus*, the aureusimines A/B, comprised of the CDP cyclo(L-Val-L-Tyr) and cyclo(L-Val-L-Phe), respectively, are involved in the regulation of bacterial virulence factors in a murine host [19]; similarly, the CDP cyclo(L-Phe-L-Pro) in* Vibrio cholerae*,* V. parahaemolyticus*, and* V. harveyi* is involved in controlling the expression of genes that are important in pathogenicity [20]. Moreover, it was reported that CDPs and DKPs may induce cell death in several cancer cell lines [21], by affecting biological processes such as microtubule polymerization; for example, cyclo(D-Tyr-D-Phe), isolated from* Bacillus *species, induced apoptosis via caspase-3 activation in the A549 pulmonary adenocarcinoma cell line [22]. In addition, it was reported that the CDPs cyclo(L-Leu-L-Pro) and* cis*-cyclo(L-Phe-L-Pro) isolated from* Lactobacillus* exhibited antiviral activity against the influenza A (H3N2) virus [23].

Although, in the context of bacteria-mammalian interaction, it has been suggested that CDPs could play an important role in bacterial pathogenesis, bacteria-host signaling, or mammalian cell growth, the mechanisms involved are unknown. Therefore, in this study, we focused on investigating the cellular effect of CDPs produced from* P. aeruginosa* strain PAO1, a pathogenic bacterium in humans that is capable of secreting the CDPs, cyclo(L-Pro-L-Tyr), cyclo(L-Pro-L-Val), and cyclo(L-Pro-L-Phe) into the culture medium (Figure 1(b)). The biological effects of these CDPs on the growth and/or pathogenesis of mammalian cells remain unknown; the* P. aeruginosa* CDPs could be involved in bacterial host colonization phenomena during disease episodes, where antiproliferative or anti-immune properties of these compounds could affect the host organism. In this regard, we employed the HeLa cervical adenocarcinoma and Caco-2 colorectal adenocarcinoma cell lines as host models in this study.

## 2. Materials and Methods

### 2.1. Chemicals and Reagents

Dulbecco's modified Eagle's medium (DMEM), fetal bovine serum (FBS), antibiotic antimycotic solution (100X) penicillin, streptomycin, and amphotericin B were purchased from Sigma-Aldrich Co. 4,6-diamidino-2-phenylindole (DAPI) and 3-(4,5-dimethylthiazol-2-yl)-2,5-diphenyltetrazolium bromide (MTT) were purchased from Sigma-Aldrich Co. Alexa Fluor 488 annexin V and the PI/dead cell apoptosis kit were obtained from Invitrogen Life Technologies (Carlsbad, CA, USA). Tissue-culture plastic ware was acquired from Corning (Tewksbury, MA, USA).

The* P. aeruginosa* CDP mix was characterized as described previously [12]. Briefly, the* P. aeruginosa* WT strain was placed in 100 mL of Luria Bertani (LB) medium and incubated for 24 h at 30°C for bacterial growth. Cell-free supernatants were prepared by centrifugation (10,000 ×g, 25°C for 10 min). The resulting supernatant was extracted twice with ethyl acetate supplied with acetic acid (0.1 mL/L). The extracts were evaporated to dryness using a rotavapor at 60°C (Buchi Co., Lawil, Switzerland). The residue was solubilized in methanol : acetonitrile (1 : 1) and analyzed by GC-MS as described [12]. The CDP mix is constituted by the cyclo(L-Pro-L-Tyr), cyclo(L-Pro-L-Val), and cyclo(L-Pro-L-Phe) in a 1 : 1 : 1 molar ratio. For dose-response assays, the CDP mix was evaporated to dryness, weighed out, and dissolved with DMSO to prepare a 100 mg/mL concentration as stock solution.

### 2.2. Cell Line Growth

The human cancer cell lines HeLa and Caco-2 were obtained from the American Type Culture Collection (ATCC, Manassas, VA, USA). Cell procedures were performed under class II biological safety cabinets. Cells were cultured in DMEM supplemented with 10% (v/v) FBS (complete medium) and 1% antibiotic (10,000 units of penicillin, 10 mg streptomycin, and 25 *μ*g of amphotericin B per mL) solution. The cultures were fed twice a week and maintained at 37°C under 80% humidity and incubated in an atmosphere of 5% CO_2_. HeLa and Caco-2 cells were collected by trypsinization using trypsin/EDTA buffered solution for 5 min at room temperature, followed by the addition of 5 mL of serum-enriched medium (CM) to stop trypsin action. After trypsinization the cells were collected and washed with CM. Finally, cells were counted in a hemocytometer chamber and incubated in fresh CM media.

### 2.3. Cell Viability Assay

Cell viability was determined by the MTT colorimetric method using thiazolyl blue tetrazolium bromide (Sigma-Aldrich Co). Briefly, HeLa and Caco-2 cells were seeded in 96-well flat-bottomed plates at a density of 3 × 10^4^ cells per well in 200 *μ*L of CM and incubated for 24 h at 37°C as described above. Then, the medium was removed and replaced with new CM or serum-free medium (SS). Then, cells were incubated with CDP mix solution at indicated concentration. Cells were incubated for another 24 h at 37°C. To determine cell viability, 10 *μ*L of 5 mg MTT per mL in PBS was added to each well and incubated for 4 h at 37°C. Finally, 100 *μ*L of 2-propanol/1 M HCl (19 : 1 v/v) was added to dissolve the formazan crystals. Absorbance measurements were conducted utilizing a microplate spectrophotometer (IMarK Microplate Reader, BIO-RAD, Hercules, CA, USA) at 595 nm.

### 2.4. Necrosis and Apoptosis Assay

HeLa and Caco-2 cell lines were seeded in 96-well flat-bottomed plates at a density of 3 × 10^4^ cells per well in 200 *μ*L of CM and incubated for 24 h at 37°C. Then, cells were synchronized with SS medium for 12 h at 37°C and were incubated with different concentrations of CDPs mixture. DMSO was used as control at same concentration used to dissolve the CDP mix. To determinate the apoptotic effect, cells were collected by centrifugation at 2,000 ×g for 10 min. The pellet was suspended in 20 *μ*L of SS medium and treated with annexin V and propidium iodide (PI) (Dead Cell Apoptosis Kit; Molecular Probes, Invitrogen Life Technologies, Carlsbad, CA, USA) following the indications recommended by the manufacturer. Fluorescence was immediately quantified by flow cytometry (FC) using a BD Accuri C6 Flow Cytometer (BD Biosciences, San Jose, CA, USA). The populations of cells for each of the treatments were gated in forward scatter and side scatter dot plots to eliminate cell debris. Populations corresponding to auto- or basal-fluorescence were located in the left quadrant and cells with emission of fluorescence increasing at least one log unit value were located in the right quadrant of the dot plots. In addition, the percentage of fluorescent cells (PFC) and median fluorescence intensity (FI) were determined in monoparametric histograms of fluorescence emission obtained from the dot plots and labeled as PFC and as relative units of fluorescence. The equipment was calibrated using Spherotech 8-peak (FL1–FL3) and 6-peak (FL-4) validation beads (BD Accuri, San Jose, CA, USA). For apoptosis and necrosis assays, fluorescence for annexin V in emission fluorescence channel FL1 at 495/519 nm and for propidium iodide in the FL2 channel at 535/617 nm was monitored. A minimum of 20,000 cellular events were analyzed.

### 2.5. Cell Image Captures

HeLa and Caco-2 cells were seeded in 12-well flat-bottomed plates with sterile-covered round objects covered with collagenase at a density of 1 × 10^4^ cells per well with one mL of CM and incubated for 24 h at 37°C. Cells were incubated with serum-free medium (SS) for 12 h at 37°C and an atmosphere of 5% CO_2_ and incubated with different concentrations of the CDP mix. After 12 h of treatment, the cells were washed with PBS. Cells were fixed with paraformaldehyde (PFA at 4%) for 10 min on ice. Then, cells were incubated with DAPI (1 : 1,000) for 10 min at room temperature. Finally, cells were washed with PBS, and the cover glass was removed and placed into a holder with a drop of PBS and glycerol 1 : 1. Cultured cells were photographed using an inverted phase-contrast microscope (Carl-Zeiss HB0-50, San Diego, CA, USA) equipped with an AxioCam/Cc1 digital camera. Cultures of HeLa and Caco-2 cells were grown in CM and incubated with DAPI and visualized using a confocal microscope (Olympus FV1000, Center Valley, PA, USA). The cells were observed by fluorescence emission between 405 and 505 nm.

## 3. Results and Discussion

### 3.1. CDPs from* P. aeruginosa* Cultures Affect the Viability of Human Cancer Cell Lines

**Figure 2:**
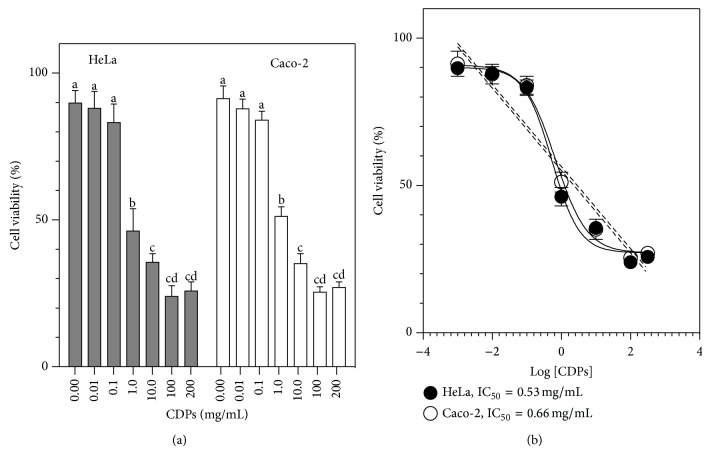
Effect of cyclodipeptides from* Pseudomonas aeruginosa* on HeLa and Caco-2 cell viability. HeLa and Caco-2 cells were incubated in CM medium containing the CDP mix for 24 h. (a) Viability was determined by the MTT assay and quantitation of fluorescence. Bars represent the mean value ± the standard error (SE) of three independent experiments. One-way analysis of variance was carried out, with Tukey's post hoc test; *n* = 6. Values for SE (*P* < 0.05) are shown in lowercase letters. (b) Nonlinear regression analysis of dose-response for the inhibition of viability by the CDP mix; 95% confidence interval, *P* < 0.001. HeLa: 50% inhibitory concentration (IC_50_) = 0.53 mg/mL; *R* ^2^ = 0.96. Caco-2: IC_50_ = 0.66 mg/mL, *R* ^2^ = 0.93.

In order to test the effect of CDPs from* P. aeruginosa* on mammalian cell growth, we used the HeLa and the Caco-2 cell lines as models in this study. The HeLa cell line has been extensively employed to test anticancer drugs [24], while the Caco-2 cell line has been used to evaluate the ability of chemicals to cross the intestinal barrier and to study their transport mechanisms [25]. A mixture of CDPs, mainly comprised of cyclo(L-Pro-L-Tyr), cyclo(L-Pro-L-Val), and cyclo(L-Pro-L-Phe) in a 1 : 1 : 1 molar ratio, was isolated from the* P. aeruginosa* PAO1 strain, grown on Luria Bertani broth. The CDP mix was applied in a dose-dependent manner to human cells grown in CM medium. The results obtained showed that CDPs caused a decrease in the viability of HeLa and Caco-2 cells in a dose-dependent manner, cell cultures exhibiting 75% dead cells following treatment with the CDP mix at 100 mg/mL (Figure 2(a)). The 50% inhibitory concentration (IC_50_) for the CDP mix from the PAO1 strain was 0.53 and 0.66 mg/mL, for HeLa and Caco-2 cells, respectively (Figure 2(b)). Although CDPs incubated in serum-free (SS) medium showed slight differences in activity compared to those incubated in serum-containing (CM) medium, these differences were not significant (see Figure S1 in Supplementary Material available online at http://dx.doi.org/10.1155/2015/197608). These findings indicate that the CDP mix from* P. aeruginosa* inhibited the viability of HeLa and Caco-2 cells and that this effect was independent of the presence or absence of serum.

**Figure 3:**
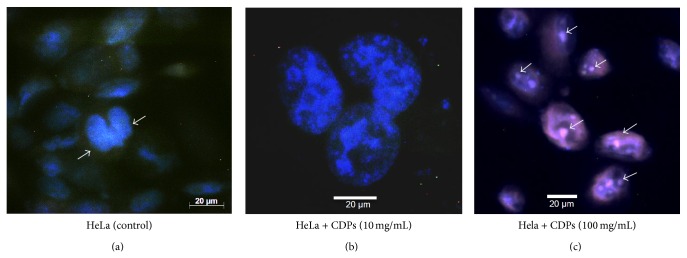
Morphological changes in HeLa cells, induced by cyclodipeptides from* Pseudomonas aeruginosa*. ((a), (b)) Images of HeLa cells were taken under phase-contrast confocal microscopy following treatment with the* P. aeruginosa* CDP mix for 24 h and staining with DAPI. Images of cells were taken at 20x magnification (a) or 40x magnification (b). (c) Assessment of nuclear condensation by DAPI staining of cells treated with* P. aeruginosa* PAO1 CDPs (20x magnification). After treatment, the number of apoptotic nuclei was increased and more nuclear condensation was observed in cultures of both cell lines, in comparison with untreated controls. Arrows indicate apoptotic nuclei.

Microscopic observation of cells following treatment with the CDP mix and staining with DAPI showed that while HeLa cells treated with DMSO solvent alone did not exhibit nuclear DNA fragmentation (Figure 3(a)), HeLa cells did exhibit nuclear DNA fragmentation after treatment with the CDP mix at a concentration of 10 mg/mL for 24 h (Figure 3(b)); in addition, apoptotic bodies were clearly visible in cells treated with CDPs (Figure 3(c)). These results indicate that the CDP mix from* P. aeruginosa* produced a decrease in cell viability by means of a mechanism of DNA fragmentation.

### 3.2. Inhibition of HeLa and Caco-2 Cell Viability by CDPs from* P. aeruginosa* Involves an Apoptotic Pathway

**Figure 4:**
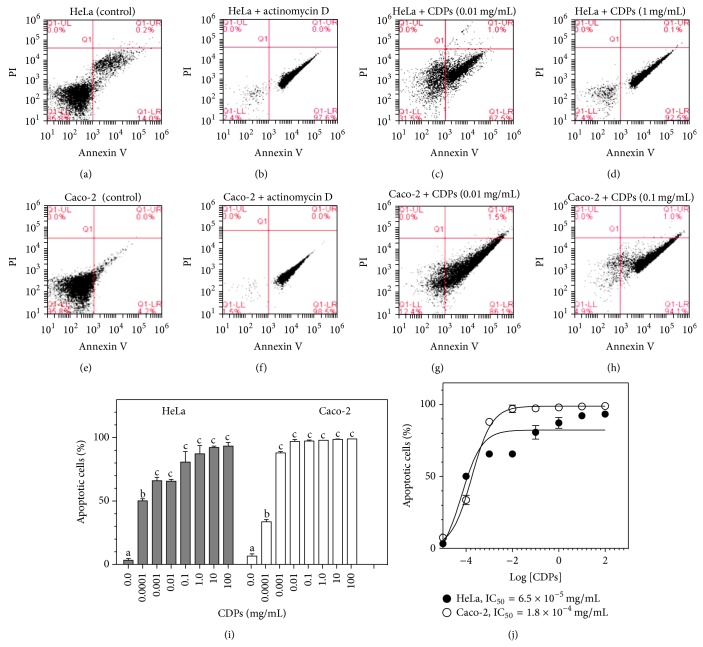
Induction of apoptosis in HeLa and Caco-2 cells by cyclodipeptides from* Pseudomonas aeruginosa*. HeLa and Caco-2 cells were incubated in CM medium after treatment with the CDP mix for 24 h. HeLa cells were stained with annexin V and propidium iodide and analyzed by flow cytometry. ((a)–(h**)**) Schematic diagrams of dot plots, showing the quadrant divisions for the determination of apoptosis using the annexin V and PI probes. The populations of cells for each of the treatments were gated in the forward scatter and side scatter analyses, in order to eliminate dead cells and cell debris. Populations corresponding to autofluorescence or basal fluorescence are located in the lower-left quadrants. Cells with increased fluorescence emission of at least one log unit are located in the lower-right quadrants. The percentage of fluorescent cells is indicated in the dot plots. HeLa cell treatment: (a) DMSO (0.05%; negative control); (b) actinomycin D (50 mg/mL; positive control); (c) 0.01 mg/mL CDP mix; (d) 1.0 mg/mL CDP mix. Caco-2 cell treatment: (e) DMSO (0.05%; negative control); (f) actinomycin D (50 mg/mL; positive control); (g) 0.01 mg/mL CDP mix; (h) 0.1 mg/mL CDP mix. (i) Dose-response plot of apoptotic cell induction by CDP treatment. Percentages of fluorescent cells, determined from dot plots, were used in this analysis. Bars represent the mean value ± the standard error (SE) of three independent experiments. One-way analysis of variance was carried out, with Tukey's post hoc test; *n* = 6. Values for SE (*P* < 0.05) are shown in lowercase letters. (j) Nonlinear regression analysis of dose-response for the induction of apoptosis by the CDP mix; 95% confidence interval, *P* < 0.001. HeLa: 50% inhibitory concentration (IC_50_) = 6.5 × 10^−5 ^mg/mL; *R* ^2^ = 0.92. Caco-2: IC_50_ = 1.8 × 10^−4 ^mg/mL; *R* ^2^ = 0.99.

**Figure 5:**
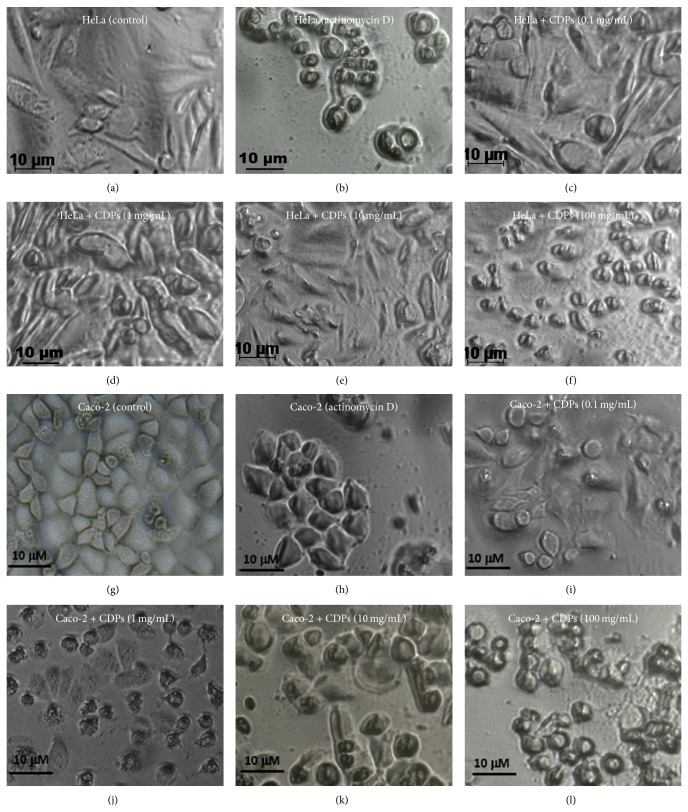
Apoptotic morphological changes in HeLa and Caco-2 cells induced by cyclodipeptides from* Pseudomonas aeruginosa*. Images of cells were taken under phase-contrast inverted microscopy after treatment with the* P. aeruginosa* CDP mix for 24 h. HeLa cell treatment: (a) DMSO (0.05%; negative control); (b) actinomycin D (50 mg/mL; positive apoptosis control); ((c)–(f)) CDP mix at a concentration of 0.1, 1.0, 10, and 100 mg/mL, respectively. Caco-2 cell treatment: (g) DMSO (0.05%; negative control); (h) actinomycin D (50 mg/mL; positive apoptosis control); ((i)–(l)) CDP mix at a concentration of 0.1, 1.0, 10, and 100 mg/mL, respectively.

In order to identify the mechanism underlying the decrease in viability of HeLa and Caco-2 cells due to CDP treatment, flow cytometric analysis was carried out using markers for apoptosis (annexin V) or necrosis (propidium iodide; PI), with cultures of both cell lines, after their treatment with the CDP mix. The value for the percentage of fluorescent cells (PFC), corresponding to cells that were positive for the annexin V marker, was ≤14% for negative controls of both HeLa and Caco-2 cells (Figures 4(a) and 4(e)), but ≥97% for both cell lines when actinomycin D was used as an apoptosis inducer (Figures 4(b) and 4(f)). Importantly, HeLa and Caco-2 cells treated with the* P. aeruginosa* CDP mix at a concentration of 0.01 mg/mL showed PFC values ≥50% for the annexin V marker (Figures 4(c) and 4(g); lower-right quadrants), increasing to ≥90% when treated with 1 mg/mL and 0.1 mg/mL CDP mix (Figures 4(d) and 4(h); resp.); however, at higher concentrations of the CDP mix, the PFC values did not show a further significant increase (Figure 4(i)). With respect to PI staining, to identify necrosis in HeLa and Caco-2 cell cultures, positive cells were not detected under the same CDP-treatment conditions (Figures 4(c), 4(d), 4(g), and 4(h); upper-right quadrants). The IC_50_ CDP-mix doses for apoptosis of HeLa and Caco-2 cells, after treatment for 24 h, were calculated as 6.5 × 10^−5^ mg/mL and 1.8 × 10^−4^ mg/mL, respectively (Figure 4(j)). These findings indicate that the CDP mix from* P. aeruginosa* caused inhibition of the viability of these two cancer cell lines via an apoptotic mechanism, in a dose-dependent manner. Interestingly, the CDP concentration for apoptosis induction in HeLa cells was 30-fold lower than for Caco-2 cells (Figure 4(j)). In addition, the CDP concentration for apoptosis induction was two log units lower than that for nonapoptotic cell death in both the cancer cell lines tested. These findings were confirmed by microscopic observation of cells; HeLa and Caco-2 cells exhibited apoptotic cell morphology following treatment with the CDP mix, at a concentration of 0.1 mg/mL (Figure 5), and similar morphology was observed following treatment with actinomycin D (apoptosis control; Figures 5(b) and 5(h)). These results indicate that CDPs from* P. aeruginosa* caused a decrease in the cell viability of HeLa and Caco-2 cell lines by means of a mechanism that involves apoptotic pathways.

These two cell lines are the most commonly used for testing drugs, for which details of molecular mechanisms of transport and cell signaling, among other biological processes, have been described. It is noteworthy that similar compounds from other organisms induce apoptosis in different cell lines, for example, cyclo(L-Pro-L-Tyr) and cyclo(L-Pro-L-Phe) isolated from* Bacillus* species. Although these CDPs were not tested for their effects on mammalian cell viability, they provoked a slight decrement in the phosphorylation of the AKT1 serine/threonine kinase at a concentration of 0.01 mg/mL in U-87 MG cells from human glioblastoma, where AKT1 inactivation is an important event that leads to apoptosis [26, 27]. The quest for novel molecules with properties involved in the control of cancer cell growth is a scientific field in growing demand [28]. Natural molecules with antiproliferative activity are considered more specific for their target than synthetic molecules; one of the probable reasons for this is that molecules produced from biological organisms such as* P. aeruginosa* tend to have more chiral centers, which give them stereochemical specificity [29]. The synthetic CDP cyclo(L-Phe-L-Pro) is capable of inducing apoptosis via cleavage of poly(ADP-ribose) polymerase in a caspase-dependent manner; however, treatment with 5 mM CDP led to apoptosis in only ≤18% of cells after 72 h [17]. In contrast, in this study, CDPs from* P. aeruginosa* induced apoptosis at a lower concentration (~0.04 mM CDP mix), in 67% and 86% of HeLa and Caco-2 cells, respectively, after 24 h. Our results indicate that the CDP mix from* P. aeruginosa* is more active in inducing cell death, leading us to suggest that the CDPs from* P. aeruginosa* could constitute a novel factor that plays important roles in the steps leading to colonization by* P. aeruginosa* of its host; these roles probably result in the inhibition of cellular growth or affect the viability of immunoprotective cells. Further investigations are necessary in order to determine the molecular target(s) of the CDPs from* P. aeruginosa* that induces apoptosis in mammalian cells.

In conclusion, this study indicates that a CDP mix composed of cyclo(L-Pro-L-Tyr), cyclo(L-Pro-L-Val), and cyclo(L-Pro-L-Phe), isolated from the* P. aeruginosa* PAO1 strain, promotes cell death of HeLa and Caco-2 cell cultures in a dose-dependent manner and suggesting an apoptotic pathway as the mechanism underlying the inhibition of cell proliferation.

## Supplementary Material

Figure S1: Effect of cyclodipeptides from *Pseudomonas aeruginosa* on HeLa and Caco-2 cell viability. HeLa and Caco-2 cells were incubated serum-free medium (SS) containing the CDP mix for 24 h. (A) Viability was determined by the MTT assay and quantitation of fluorescence. Bars represent the mean value ± the standard error (SE) of three independent experiments. One-way analysis of variance was carried out, with Tukey's post-hoc test; *n* = 6. Values for SE (*P* < 0.05) are shown in lower-case letters. (B) Nonlinear regression analysis of dose-response for the inhibition of viability by the CDP mix; 95% confidence interval, *P* < 0.001. HeLa: 50% inhibitory concentration (IC_50_) = 0.49 mg/mL; *R* ^2^ = 0.96. Caco-2: IC_50_ = 0.75 mg/mL, *R* ^2^ = 0.93.
