# Supplementary figures and images for: Cytotoxicity of Cyclodipeptides from Pseudomonas aeruginosa PAO1 Leads to Apoptosis in Human Cancer Cell Lines
Source: Biomed Res Int. 2015 Mar 2;2015:197608. doi: 10.1155/2015/197608 (PMC4363556; doi:10.1155/2015/197608)

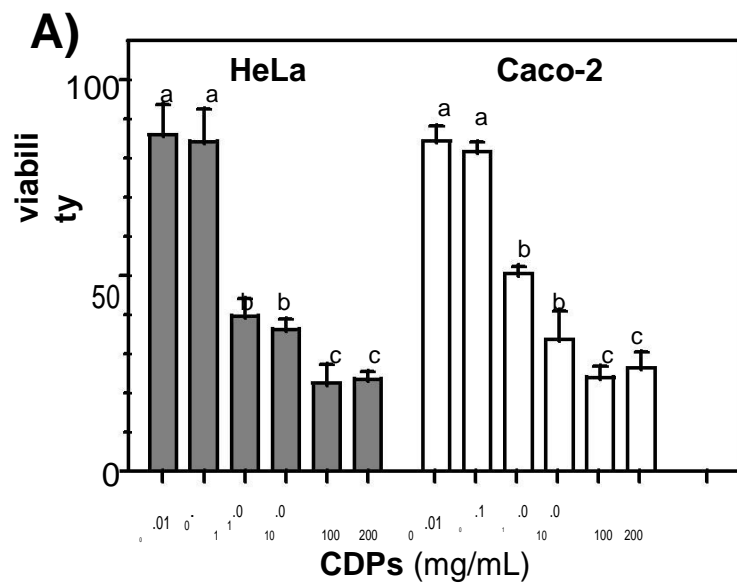

1

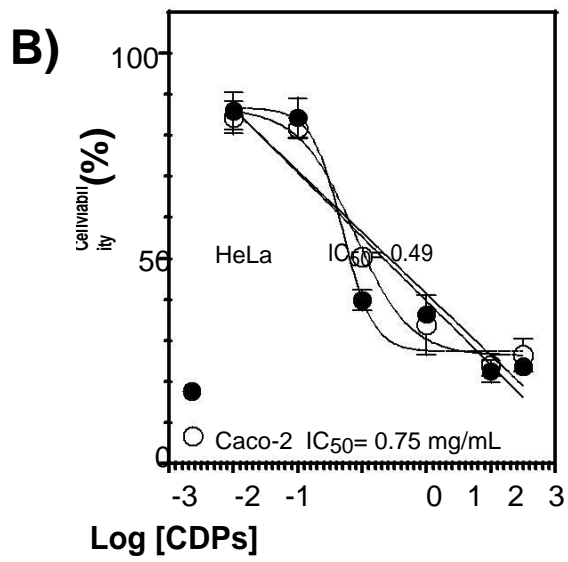

Figure S1 (supplementary material).

Supplement: Supplementary file 1 — Figure S1: Effect of cyclodipeptides from Pseudomonas aeruginosa on HeLa and Caco-2 cell viability. HeLa and Caco-2 cells were incubated serum-free medium (SS) containing the CDP mix for 24 h. (A) Viability was determined by the MTT assay and quantitation of fluorescence. Bars represent the mean value ± the standard error (SE) of three independent experiments. One-way analysis of variance was carried out, with Tukey's post-hoc test; n = 6. Values for SE (P < 0.05) are shown in lower-case letters. (B) Nonlinear regression analysis of dose-response for the inhibition of viability by the CDP mix; 95% confidence interval, P < 0.001. HeLa: 50% inhibitory concentration (IC50) = 0.49 mg/mL; R 2 = 0.96. Caco-2: IC50 = 0.75 mg/mL, R 2 = 0.93. [file 197608.f1.pdf]
